# Numerical Convergence Analysis of the Frank–Kamenetskii Equation

**DOI:** 10.3390/e22010084

**Published:** 2020-01-09

**Authors:** Matthew Woolway, Byron A. Jacobs, Ebrahim Momoniat, Charis Harley, Dieter Britz

**Affiliations:** 1 School of Computer Science and Applied Mathematics, University of the Witwatersrand, Johannesburg, Private Bag 3, Wits 2050, South Africa; matthew.woolway@wits.ac.za (M.W.); charis.harley@gmail.com (C.H.); 2 Wits Institute of Data Science (WIDS), University of the Witwatersrand, Johannesburg, Private Bag 3, Wits 2050, South Africa; 3 Department of Mathematics and Applied Mathematics, University of Johannesburg, P.O. Box 524, Auckland Park 2006, South Africa; ebrahim.momoniat@gmail.com; 4 Department of Chemistry, Aarhus University, Langelandsgade 140, 8000 Århus C, Denmark; britz@chem.au.dk

**Keywords:** Frank-Kamenetskii, crank-nicolson, high-order finite differences, convergence analysis

## Abstract

This work investigates the convergence dynamics of a numerical scheme employed for the approximation and solution of the Frank–Kamenetskii partial differential equation. A framework for computing the critical Frank–Kamenetskii parameter to arbitrary accuracy is presented and used in the subsequent numerical simulations. The numerical method employed is a Crank–Nicolson type implicit scheme coupled with a fourth order spatial discretisation as well as a Newton–Raphson update step which allows for the nonlinear source term to be treated implicitly. This numerical implementation allows for the analysis of the convergence of the transient solution toward the steady-state solution. The choice of termination criteria, numerically dictating this convergence, is interrogated and it is found that the traditional choice for termination is insufficient in the case of the Frank–Kamenetskii partial differential equation which exhibits slow transience as the solution approaches the steady-state. Four measures of convergence are proposed, compared and discussed herein.

## 1. Introduction

Thermal ignition has been of a topic of interest for many years, and continues to be an area of scientific relevance. The development of such explosions and solutions dependent upon various geometries present numerous challenges. Exothermic reactions are rife within the real world with many pertaining to sudden increases in pressure confined to physical boundaries. The impact of such constraints forces thermal reactions, heating systems to the point of explosion or steady-state.

This initial steady-state was originally described by Frank–Kamenetskii [1], extended further in 1969 to the time development of a thermal reaction [2]. The governing equation for this process under alternative notation is [2,3],

(1) $$\frac{\partial T}{\partial \tau }=K\left(\frac{\partial^{2}T}{\partial X^{2}}+\frac{D-1}{X}\frac{\partial T}{\partial X}\right)+\frac{Qz}{c}\exp\left(\frac{-E}{RT}\right),$$

where *X* the spatial variable, *T* is the temperature, τ the time, *K* the thermal diffusivity (m${}^{2}$ s${}^{-1}$), *c* the specific heat capacity (J kg${}^{-1}$ K${}^{-1}$), *Q* the heat of the reaction (J kg${}^{-1}$), *z* the pre-exponential Arrhenius parameter (s${}^{-1}$), *E* the activation energy of the reaction and *R* the gas constant. The spatial variable describes the distance from the centre of the body for either (D=1) a slab with thickness 2a and large length, (D=2) a cylinder with radius *a* and large length or (D=3) a sphere of radius *a*. Thus *D* specifies the geometry in which the reaction is taking place. Boundary conditions are provided as

(2) $$\tau \leq 0:T=T_{0}\;\mathrm{for}\mathrm{all}X,$$

(3) $$\tau >0:T\left(X=a\right)=T_{0},$$

(4) $$\;\frac{\partial T(0,\tau )}{\partial X}=0.$$

Other boundary conditions have been employed (see [3] and references therein) however we will employ those provided by ((3) and (4)).

Normalisations are as follows [3]

(5) $$\begin{array}{cc} u & =\frac{1}{\epsilon }\left(\frac{T-T_{0}}{T_{0}}\right),\\ t & =\frac{k}{a^{2}}\;\tau ,\\ x & =X/a\;. \end{array}$$

The Frank–Kamenetskii parameter δ is defined as [1,2,4],

(6) $$\delta =\frac{a^{2}Qz}{Kc\epsilon T_{0}}\exp\left(-\epsilon^{-1}\right)\;,$$

where

(7) $$\epsilon \equiv \frac{RT_{0}}{E},$$

expresses the rate of the reaction at ambient temperature $T_{0}$ and is often ≪1.

This results in the dimensionless partial differential equation (PDE)

(8) $$\frac{\partial u}{\partial t}=\frac{\partial^{2}u}{\partial x^{2}}+\frac{k}{x}\frac{\partial u}{\partial x}+\delta \exp\left(\frac{u}{1+\epsilon u}\right)\;,$$

where k=D−1. The Frank–Kamenetskii parameter is of considerable importance given that at values below the critical value thereof, $\delta_{cr}$, a steady-state solution is reached for respective geometry and boundary conditions, while a thermal explosion presents itself for values above it.

The Frank–Kamenetskii partial differential equation (FKPDE) has received a great deal of attention in recent years both in terms of computational and analytical work. Momoniat [5] obtained new solutions to the FKPDE via a non-classical symmetry approach. Numerical solutions have been obtained by Britz et al. [3], Harley [6], Momoniat [7] and Soliman [8] via various numerical techniques. Motsa and Sibanda [9] present a quasi-linearisation technique for nonlinear differential equations with higher-order convergence which is applied to the FKPDE. The presented method verifies the critical δ value of 2 obtained by Britz et al. [3] for the cylindrical geometry and activation parameter ϵ=0.

It has been observed that numerical solutions to the steady equation [3] and those obtained from the transient equation [7] exhibited some discrepancies. In this work, we consider the convergence performance of four terminating criteria with high-order spatial discretisations for a Crank–Nicolson time-marching scheme, used for direct calculation of the steady-state solution. The motivation behind this work comes from uncertainty regarding the results obtained in [7] which centred around the use of higher-order discrete approximations. In particular, although central-difference five-point approximations to spatial derivatives were applied in the bulk of the propagation matrix, lower-order approximations were used at the boundaries. We now propose the use of asymmetric higher-order approximations at these points as a means of maintaining the order accuracy of the scheme. This is an important consideration given that due to symmetry, the temperature profile depicts the behaviour of the temperature at the centre of the vessel on one of the boundaries. Furthermore, we seek to clarify some issues surrounding the steady-state solution of the FKPDE and the termination criterion employed in the numerical schemes considered. We note here that the term ‘convergence’ is traditionally used in numerical analysis to describe the convergence of a numerical approximation to the true underlying exact solution in the limit of some discretisation parameters. In this work we interrogate the convergence of the numerical solution toward the steady-state solution for long time simulations and specifically the termination criteria employed dictating this convergence to some tolerance.

The FKPDE exhibits a vast range of dynamics given the parameters, however, the focus of this research is a comparative analysis of termination criteria which is conducted across three geometries (k=0 for the large slab geometry, k=1 for the cylindrical geometry and k=2 for the spherical geometry) while taking the activation parameter ϵ to be 0. Then Equation (8) reduces to

(9) $$\frac{\partial u}{\partial t}=\frac{\partial^{2}u}{\partial x^{2}}+\frac{k}{x}\frac{\partial u}{\partial x}+\delta \;e^{u},$$

in which *u* is the normalised temperature, *t* the normalised time and *x* the spatial variable where 0≤x≤1 and δ is defined as the Frank–Kamenetskii parameter. We impose the boundary conditions

(10) $$\begin{array}{cc} \frac{\partial u(0,t)}{\partial x} & =\;0, \end{array}$$

(11) $$\begin{array}{cc} u(1,t) & =\;0\;, \end{array}$$

and initial condition

(12) u(x,0)=0.

The remainder of the paper is structured as follows. Section 2 discusses the candidacy of various methodologies for the solution of the Frank–Kamenetskii equation. Section 3 provides an analysis of the critical value of the Frank–Kamenetskii equation. Section 4 introduces the Crank–Nicolson scheme used in the structuring of a finite difference scheme for the solution of the FKPDE. Section 5 proposes several terminating criteria used for the numerical experiments conducted, with the results of these criteria discussed in Section 6. Finally, conclusions are drawn in Section 7.

## 2. Candidate Methodologies

This section presents some additional details of potential numerical methodologies amenable to the numerical solution of the Frank–Kamenetskii equation as well as supporting reasoning behind the selection of the implemented numerical method.

The Lawson-Euler [10] methodology implemented by Momoniat in [7] essentially treats the linear part of the Frank–Kamenetskii equation exactly by way of an integrating factor. The truncation error of the Lawson-Euler scheme is $O\left(▵t\right)+O\left(▵x^{4}\right)$.

By replacing the time integration procedure of the Lawson-Euler scheme with a fourth-order Runge-Kutta scheme one may obtain (details of the implementation may be found in [5]) a scheme with truncation error of $O\left(▵t^{4}\right)+O\left(▵x^{4}\right)$. This scheme enjoys a higher accuracy in the temporal discretisation at the cost of four additional evaluations at each time step.

One possible solution methodology distinct from finite difference schemes are pseudo-spectral methods. This class of methodologies is attractive due to their geometric convergence and hence it is generally acceptable to use fewer gridpoints while still obtaining accurate solutions. The applicability of a Chebyshev collocation spatial discretization was explored and it was found that due to the dense differentiation matrix the method can become computationally expensive. Moreover, as the number of collocation points increases the truncation error decreases but round-off errors accumulate and dominate the error [11,12]. In addition to the accumulation of these errors, instabilities in the scheme require an appropriately small choice of Δt which hinders the convergence of the method.

The above methodologies produce time dynamic numerical simulations to the Frank–Kamenetskii equation. Given the nature of the equation and the potential interest in the steady-state solution to the equation, an accurate numerical solution to the steady solution may be readily obtained using the shooting method with Richardson’s extrapolation, as conducted by Britz et al. [3]. Our interest is in the behaviour of time-marching schemes, and hence this method is not of interest in this work.

The discussed methodologies were implemented and compared for the Crank–Nicolson scheme (the details of which are discussed in Section 4.2). It was found that unconditional stability afforded by the Crank–Nicolson scheme rendered it most amenable to the long-time simulation of the Frank–Kamenetskii equation. Moreover the Crank–Nicolson implementation presented in this work enjoys $O\left(▵t^{2}\right)+O\left(▵x^{4}\right)$ accuracy.

## 3. Frank–Kamenetskii Parameter δ

**Figure 1:**
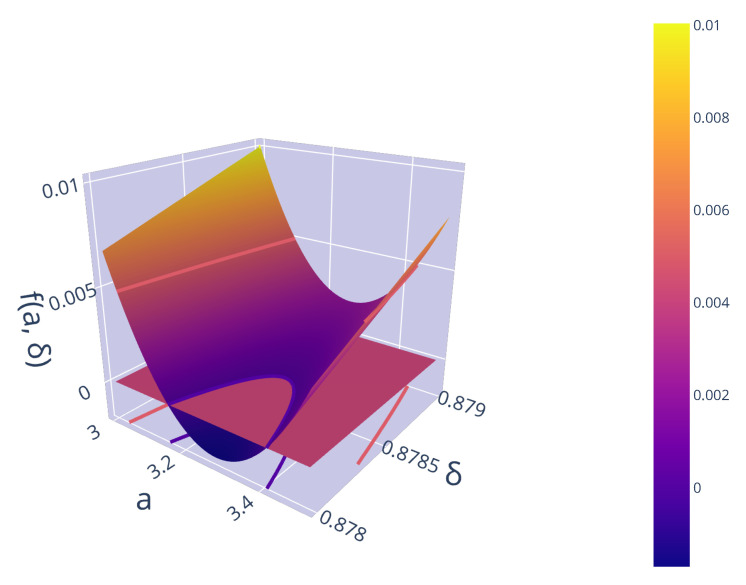
Parabolic nature of δ near criticality.

In this section we will begin by establishing a robust approach for computing the critical value of the Frank–Kamenetskii parameter δ, below which steady solutions are obtained, with arbitrary precision, to be used for the numerical simulations to follow in subsequent Sections. Beginning with the steady-state solution derived by Frank–Kamenetskii [2,4] for the large slab geometry,

(13) $$U\left(x\right)=\ln\left(\frac{a}{\cosh^{2}\left(x\sqrt{\frac{a\delta }{2}}\right)}\right),$$

where *a* is the solution to the auxiliary equation

(14) $$a=\cosh^{2}\left(\sqrt{\frac{a\delta }{2}}\right),$$

we define

(15) $$f\left(a,\delta \right)=\cosh^{2}\left(\sqrt{\frac{a\delta }{2}}\right)-a,$$

so that (a,δ) pairs that satisfy f(a,δ)=0 are of particular interest. Figure 1 visualises this function close to the critical value of δ, with a zero plane to illustrate the parabolic nature of δ near the criticality. Rearranging we find that

(16) $$\delta =\frac{2\cosh^{-1}{\left(\sqrt{a}\right)}^{2}}{a},$$

satisfies f(a,δ)=0, parameterising the parabolic shape seen in Figure 1. The value of *a* which maximises δ is defined in terms of the roots of a complicated polynomial, which is unfortunately intractable analytically. A more pragmatic approach is to maximise Equation (16) by solving for *a* when $\frac{d\delta }{da}=0$, given explicitly by

(17) $$\frac{d\delta }{da}=\frac{2\cosh^{-1}\left(\sqrt{a}\right)}{\sqrt{\sqrt{a}-1}\sqrt{\sqrt{a}+1}a^{3/2}}-\frac{2\cosh^{-1}{\left(\sqrt{a}\right)}^{2}}{a^{2}}=0.$$

Finding the root of this function can be done numerically to arbitrary accuracy. Applying the bisection method with a tolerance of $10^{-16}$ (machine precision) initially on the domain a∈[3,4] we obtain in 54 iterations,

(18) δ=0.8784576797812904,

corresponding to

(19) a=3.2767175312280727.

Substituting the above values for *a* and δ into Equation (13) we can find the corresponding left-hand boundary value to be

(20) U(0)=1.1868421686343897.

We note here that the critical value for δ was investigated by Britz et al. [3] and was accurately found to eight significant figures utilising the shooting method on the steady equation which is widely agreed upon in the literature. The result obtained in (18) is accurate to seventeen significant figures and the methodology does not require the discretisation of the differential equation, although it does require the exact steady solution. The exact critical value for δ in the cylindrical case is readily found to be 2 from the exact solution presented by Harley [6], and in the spherical case the critical value of δ=3.3219921, as reported in [3], is used.

## 4. Crank–Nicolson

The Crank–Nicolson scheme is an average of the explicit and implicit finite difference spatial discretisation. The numerical scheme enjoys the stability properties of the implicit finite difference discretisation but also exhibits a higher order temporal accuracy than either the explicit or implicit finite difference methods. The numerical implementation selected for the experiments conducted in Section 6 is a Crank–Nicolson type scheme with fourth order spatial discretisations which ensures the scheme exhibits sufficient accuracy so as not to obfuscate the convergence behaviour.

### 4.1. Spatial Discretisation

In the following sections, our space domain is divided into *N* intervals of size 1/N and we make use of fourth-order discretisations for the spatial derivatives. The central difference discretisation is given as

(21) $$\begin{array}{cc} {\left.\frac{\partial u}{\partial x}\right|}_{x=x_{i}} & \approx \frac{u_{i-2}-8u_{i-1}+8u_{i+1}-u_{i+2}}{12▵x}+O\left({▵x}^{4}\right),\\ \; & \;\;\;\;\;\;i=2,3,\ldots ,N-2\;. \end{array}$$

and

(22) $$\begin{array}{cc} {\left.\frac{\partial^{2}u}{\partial x^{2}}\right|}_{x=x_{i}} & \approx \frac{-u_{i-2}+16u_{i-1}-30u_{i}+16u_{i+1}-u_{i+2}}{12{▵x}^{2}}+O\left({▵x}^{4}\right),\\ \; & \;\;\;\;\;\;i=2,3,\ldots ,N-2\;. \end{array}$$

Given the symmetric nature of the geometry under consideration we have employed a Neumann boundary condition at x=0, which implies

(23) $$u_{-i}=u_{i},\;i=1,2\;.$$

The left hand boundary approximations thus become

(24) $${\left.\frac{\partial u}{\partial x}\right|}_{x=x_{0}}=0\;,$$

and

(25) $${\left.\frac{\partial^{2}u}{\partial x^{2}}\right|}_{x=x_{0}}\approx \frac{-30u_{0}+32u_{1}-2u_{2}}{12{▵x}^{2}}+O\left({▵x}^{4}\right)\;,$$

and at x=▵x

(26) $${\left.\frac{\partial u}{\partial x}\right|}_{x=x_{1}}\approx \frac{-8u_{0}+u_{1}+8u_{2}-u_{3}}{12▵x}+O\left({▵x}^{4}\right)\;.$$

and

(27) $${\left.\frac{\partial^{2}u}{\partial x^{2}}\right|}_{x=x_{1}}\approx \frac{16u_{0}-31u_{1}+16u_{2}-u_{3}}{12{▵x}^{2}}+O\left({▵x}^{4}\right)\;.$$

We note here that the Neumann boundary condition discretisations (23) are second-order accurate hence the approximations at the boundaries also have second-order accuracy. At $x_{N-1}=1-▵x$ we cannot invoke any physical symmetry, nor can we apply (22) directly since we will require values outside of the grid space. Here we employ a fifth-order asymmetric six-point approximation as follows,

(28) $$\begin{array}{cc} {\left.\frac{\partial u}{\partial x}\right|}_{x=x_{N-1}} & \approx \frac{-u_{N-4}+6u_{N-3}-18u_{N-2}+10u_{N-1}+3u_{N}}{12▵x}\\ \; & \;\;\;\;\;\;\;\;\;+O({▵x}^{4})\;, \end{array}$$

and

(29) $$\begin{array}{cc} {\left.\frac{\partial^{2}u}{\partial x^{2}}\right|}_{x=x_{N-1}} & \approx \frac{u_{N-5}-6u_{N-4}+14u_{N-3}-4u_{N-2}-15u_{N-1}+10u_{N}}{12{▵x}^{2}}\\ \; & \;\;\;\;\;\;\;\;\;+O({▵x}^{4})\;, \end{array}$$

which provides better accuracy, which a fourth-order five-point approximation would not [13]. The coefficients were obtained using the Fornberg algorithm [14]. Finally at $x_{N}=1$ we impose the condition $u_{N}=0$, noting that no derivative is needed there for the time march or direct solution.

We discretise Equation (9), using Equations (21), (22) and (24)–(29) to obtain, in vector form,

(30) $$\frac{du}{dt}=\mathrm{Au}+S,$$

where $S\equiv {\left[\delta \;e^{u_{0}},\delta \;e^{u_{1}},\cdots ,\delta \;e^{u_{N-1}}\right]}^{T}$, $A=\frac{k}{x}B+C$ with

(31) $$B=\frac{1}{12▵x}\left[\begin{array}{ccccccccc} 0 & 0 & 0\\ -8 & 1 & 8 & -1\\ 1 & -8 & 0 & 8 & -1\\ \; & \ddots & \; & \ddots \\ \; & \; & \; & \; & 1 & -8 & 0 & 8 & -1\\ \; & \; & \; & 0 & -1 & 6 & -18 & 10 & 3 \end{array}\right]\;.$$

and

(32) $$C=\frac{1}{12{▵x}^{2}}\left[\begin{array}{ccccccccc} -30 & 32 & -2\\ 16 & 31 & 16 & -1\\ -1 & 16 & -30 & 16 & -1\\ \; & \ddots & \; & \ddots \\ \; & \; & \; & \; & -1 & 16 & -30 & 16 & -1\\ \; & \; & \; & 1 & -6 & 14 & -4 & -15 & 10 \end{array}\right]\;,$$

The value $u_{N}$ is set to zero because of the boundary conditions.

### 4.2. Implementation

Implementing the Crank–Nicolson scheme on Equation (30) produces

(33) $$2\frac{u^{j+1}-u^{j}}{▵t}=Au^{j}+Au^{j+1}+\delta \exp\left(u^{j}\right)+\delta \exp\left(u^{j+1}\right)\;,$$

where we note that $u^{j+1}$ denotes the next, unknown, value. Incorporating the relevant boundary conditions as discussed in Section 4.1 and given $u_{N}=0$, we can rearrange Equation (33) to construct the following matrix-vector equation

(34) $$M_{1}u^{j+1}+\delta \exp\left(u^{j+1}\right)+M_{2}u^{j}+\delta \exp\left(u^{j}\right)=0,$$

where

(35) $$M_{1}\equiv A-\frac{2}{▵t}I,$$

with I the identity matrix and

(36) $$M_{2}\equiv A+\frac{2}{▵t}I\;.$$

In this manner, we can implicitly treat the nonlinear source term resulting in a nonlinear system to be solved by Newton’s method for each iterate. To achieve this we iteratively solve the linearised system given by

(37) $${\left.J\right|}_{u^{j}}\Delta S={\left.-F\right|}_{u^{j}},$$

where S is initialised to $u^{j}$ for each application of Newton’s method and J is the Jacobian given by

(38) $$J=M_{1}+\delta \;\mathrm{diag}\left(\exp\left(u\right)\right),$$

with

(39) $$F=M_{2}u^{j}+\delta \exp\left(u^{j}\right).$$

The Newton iteration is stopped when the norm of $\Delta S<10^{-8}$, generally, 3–4 iterations is sufficient due to the quadratic convergence of Newton’s method. The truncation error associated with this Crank–Nicolson scheme is then $O\left(▵t^{2}\right)+O\left(▵x^{4}\right)$.

## 5. Convergence Criteria

In this section, we investigate discrepancies in convergence criteria and critically analyse their applicability to the numerical solution of the FKPDE for which dependency on convergence is critical in defining the barrier between stable solutions and physical blow up. A common practice within the literature is to deem that a numerical simulation has “converged” when

(40) $$∥u^{n+1}-u^{n}∥{}_{\infty }<\mathrm{tol},\;\left(\mathrm{Metric}\;1\right)$$

where tol refers to the chosen tolerance, is satisfied. Indeed, this is the criterion utilised by Momoniat [7]. The problem with Equation (40) is that if the order of the method used to determine u is $O(\Delta t^{n})$. Then Equation (40) approximately yields,

(41) $$\Delta t^{-(n+1)}<\mathrm{tol}$$

Explicitly, if tol is $10^{-6}$ then n>5. If the method used doesn’t guarantee this the termination criterion may yield early termination.

We propose that this inconsistency is attributed to the convergence condition and not the order of discretization. Rather, equations such as the Frank–Kamenetskii equation that exhibit slow temporal dynamics can trigger premature satisfaction of the above criterion. The convergence condition quoted in Equation (40) is derived from an O(Δt) approximation to $\frac{du}{dt}=0$ and is insufficient in obtaining the true steady solution. In this section, we introduce three new alternative convergence conditions in an attempt to address this early termination.

The first new criterion proposed is derived from a fourth-order backward difference approximation to the temporal derivative and is given by

(42) $$\frac{du}{dt}=\frac{3u^{j+1}+10u^{j}-18u^{j-1}+6u^{j-2}-u^{j-3}}{12\Delta t}+O\left(\Delta t^{4}\right),$$

yielding the criterion

(43) $${∥3u^{j+1}+10u^{j}-18u^{j-1}+6u^{j-2}-u^{j-3}∥}_{\infty }<\mathrm{tol}.\;\left(\mathrm{Metric}\;2\right)$$

Taking into account the slow temporal dynamics exhibited by the Frank–Kamenetskii equation, the second convergence criterion proposed is based on the spatial solution of the equation. This measure assesses how closely the current time step satisfies the steady equation and is given by

(44) $$∥Au^{j+1}+\delta \exp\left(u^{j+1}\right)∥{}_{\infty }<\mathrm{tol}.\;\left(\mathrm{Metric}\;3\right)$$

Our final proposition for convergence is comparing the numerical solution at the current time step to the exact steady-state solution. While this condition is reliant on the known exact solution to the steady-state we use this in the current study as a benchmark against which the other convergence criteria may be assessed. This condition is given by

(45) $$∥u^{j+1}-U\left(x\right)∥{}_{\infty }<\mathrm{tol},\;\left(\mathrm{Metric}\;4\right)$$

where U(x) is the exact steady solution given by Equation (13) with x∈[0,1] and discretised as previously stated.

## 6. Results

All results were obtained on a AMD Ryzen™ Threadripper™ 1950x CPU @ 3.4GHz, with 128 GB RAM @ 2400 MHz on Ubuntu 19.10 implemented in Mathematica 12.

**Figure 2:**
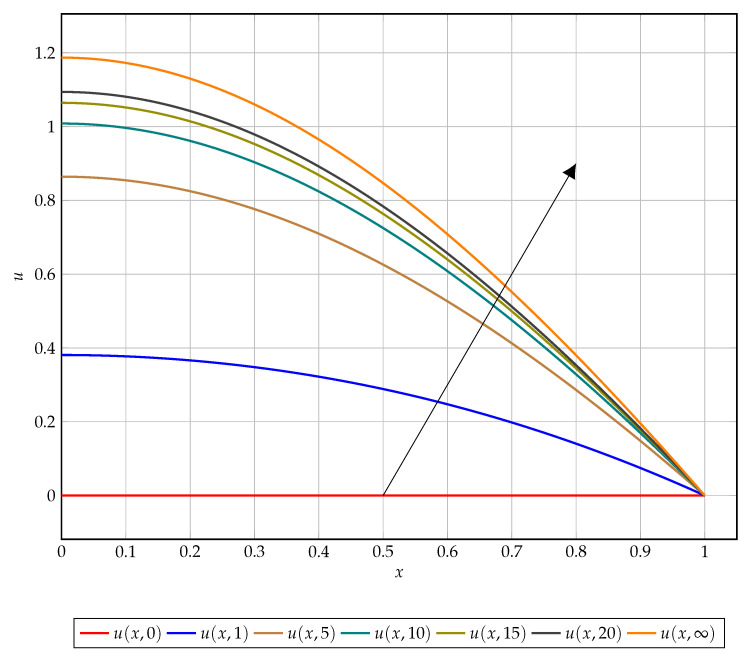
Solution plot of u(x,t) over *x* for t=0,1,5,10,15,20,∞ (corresponding to the exact steady-state solution) increasing in the direction of the arrow.

Figure 2 serves as an aid for the reader to visualise the equation dynamics, this figure illustrates the convergent dynamics as time tends to infinity. Particularly, the dynamics of the left-hand boundary value which is indicative of the overall convergence of the solution.

### 6.1. Infinite Slab Geometry (k=0)

The Crank–Nicolson scheme derived in Section 4 was implemented for the large slab geometry and each of the four terminating criteria discussed in Section 5. The obtained results for each termination criterion are given in Table 1, Table 2, Table 3 and Table 4. For consistency all experimental parameters were fixed, Δx=Δt=1/200, δ=0.8784576797812904 as described and calculated in Section 3, with only the termination criterion changing for various experiments. The tables presented below illustrate, column-wise, the tolerance for which the termination criterion is satisfied, the resulting left-hand boundary value, the given measure of convergence, number of iterations taken to satisfy that measure of convergence, absolute CPU time elapsed and finally the absolute distance between the simulated boundary and the exact steady boundary value.

Table 1 illustrates the early termination exhibited by the traditionally [7] used measure of convergence. This measure reports ‘convergence’ to $O(10^{-8})$ when the solution is only accurate up to $O(10^{-3})$ when compared to the steady-state value at the left-hand boundary.

The second convergence criterion assessed improves on the traditional selection by measuring the rate of change to $O(\Delta^{4})$. This choice again leads to premature termination of the numerical simulation as can be seen in the last row of the table, with the measure reporting convergence to $O(10^{-8})$ when the solution is only $O(10^{-4})$ accurate when compared to the steady-state value at the left-hand boundary.

The dynamics of the Frank–Kamenetskii equation give rise to extremely slowly evolving solutions. As such, the third measure of convergence is not based on measuring the rate of evolution but rather on ‘how well’ the approximate solution satisfies the steady equation. This yields a more accurate convergence criterion than the previous experiments, reporting convergence to $O(10^{-5})$ from the steady-state value at the left-hand boundary when the criterion indicates convergence to $O(10^{-8})$. We note here that in the event that the steady-state solution is unknown this convergence criterion may still be employed yielding a more robust measure of convergence when numerical solutions are evolving slowly.

Results for the final experiment are presented in Table 4. We note that columns three and six are commensurate, indicating this measure is absolute in measuring the distance of the solution from the steady-state. It is also worth noting that the infinity norm measures maximum absolute distance which, in this case, always occurs at the left-hand boundary supporting the exact agreement between columns three and six.

**Figure 3:**
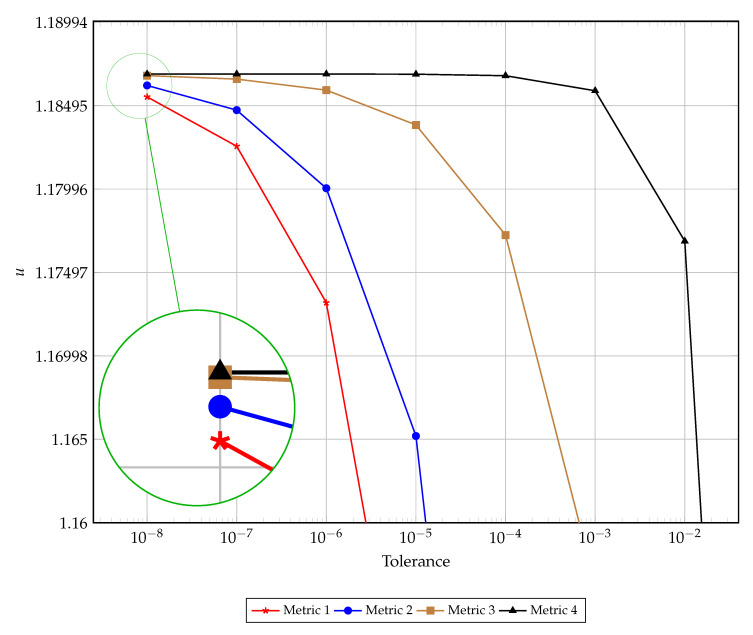
The convergence of terminating criteria over varying tolerance.

**Figure 4:**
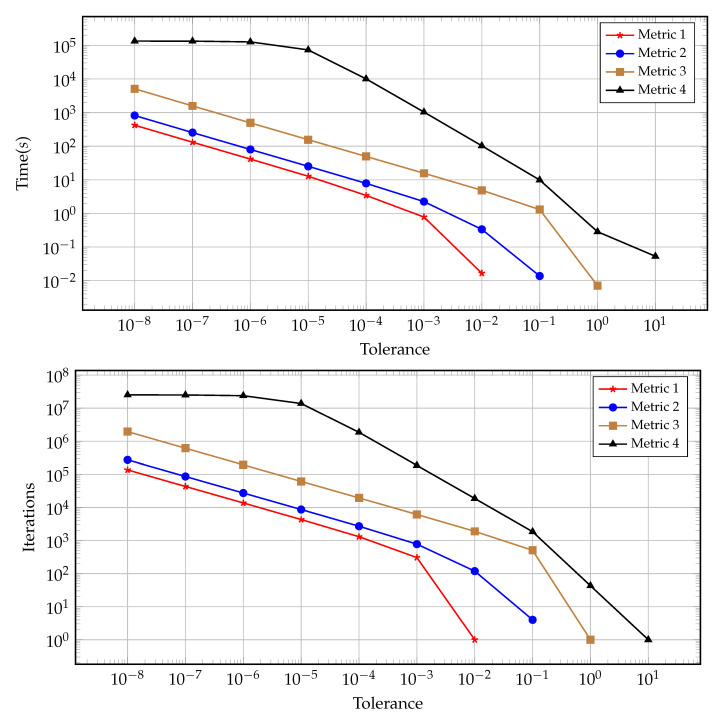
Time and iterations required over varying tolerance.

A visual comparison of the early termination is presented in Figure 3. This figure illustrates the left hand boundary value at the time of convergence as dictated by each of the four discussed metrics for convergence for varying tolerances. The results presented in this figure support the findings reported in Table 1, Table 2, Table 3 and Table 4. Figure 4 presents the computational time and number of iterations taken to reach a prescribed tolerance for each of the convergence metrics. As expected, the computational time and number of iterations are highly correlated, with small discrepancies occurring at very low tolerances due to algorithmic overhead. Interestingly the slope of the relationship between the tolerance and number of iterations is the same for metrics one through three, whereas metric four has a steeper gradient (implying the need for proportionally more iterations to satisfy the next tolerance order) for lower tolerances and a much flatter gradient for higher tolerances. Again, this is evident in Table 4 where satisfying the higher tolerances occurs after fewer iterations when compared with satisfying lower tolerances (specifically requiring 10 million iterations moving from $O(10^{-4})$ to $O(10^{-5})$ and to $O(10^{-6})$ but only 1.2 million to then move to $O(10^{-7})$ and 100 thousand iterations to get to $O(10^{-8})$).

### 6.2. Cylindrical Geometry (k=1)

We now assess the convergence of the Crank–Nicolson scheme for the cylindrical geometry. Again, for consistency all experimental parameters were fixed, Δx=Δt=1/1000, δ=2, with only the termination criterion changing for various experiments. Table 5, Table 6, Table 7 and Table 8 support the same findings as in the case of the large slab, specifically that a poor choice of convergence criterion leads to early termination of the numerical simulation.

**Figure 5:**
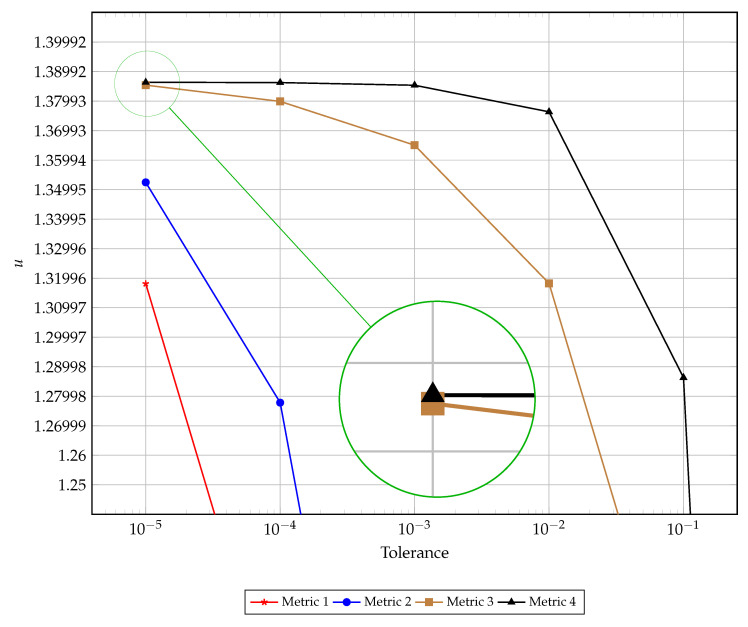
The convergence of terminating criteria over varying tolerance.

The cylindrical geometry gives rise to interesting dynamics since the critical value of δ is known exactly. This value is asymptotic, below which stable solutions exist and above which blow up occurs. The δ value used for the other geometries is approximated numerically (to high precision) and tends to the true critical value from the left which ensures stable simulations. In the cylindrical geometry, since we are exactly at the critical value, numerical simulations will cross the threshold of stable computation after about 400,000 iterations, in the current experimental setup. This necessitates the use of a lower tolerance in Figure 5 for a meaningful comparison between the candidate metrics. As before we observe metrics 1 and 2 result in premature termination of the numerical simulation, while metric 3 most accurately predicts convergence to steady-state.

### 6.3. Spherical Geometry (k=2)

**Figure 6:**
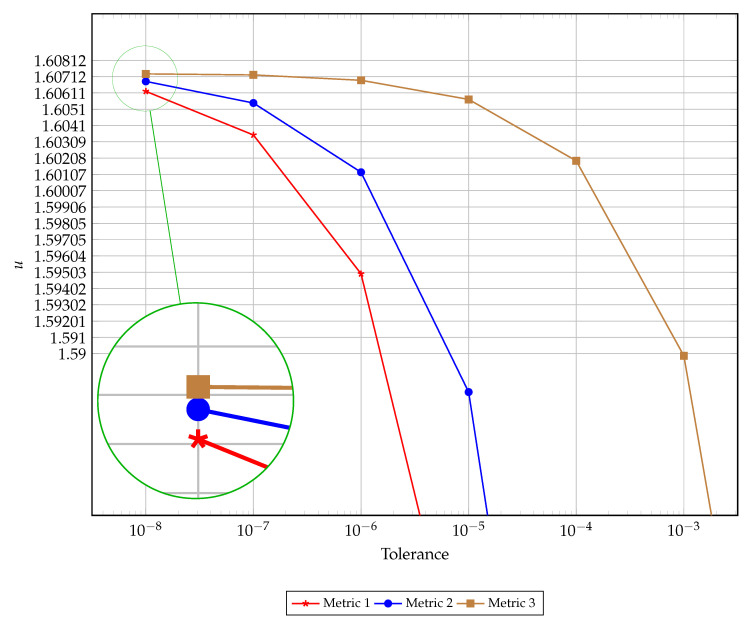
The convergence of terminating criteria over varying tolerance.

Finally, we consider the convergence dynamics for the spherical geometry. Once more, all experimental parameters were fixed, Δx=Δt=1/1000, δ=3.3219921, with only the termination criterion changing for various experiments. Table 9, Table 10 and Table 11 illustrate the convergence of the Crank–Nicolson scheme under the candidate convergence criteria. To the best of the authors’ knowledge no exact solution exists for the spherical geometry steady equation, as such we are only able to present results for the first three metrics. The final column in Table 9, Table 10 and Table 11 present the absolute distance between the transient solution and the steady solution reported by Britz et al. [3]. Figure 6 illustrates the critical temperature at convergence as dictated by the various metrics for varying tolerances.

### 6.4. Discussion

The results presented in this section highlight the ‘subjectivity of convergence’ introduced by the choice of criterion. Since all experiments were conducted with the same discretisation parameters, the number of iterations taken to reach convergence yield an absolute measure of premature termination. When comparing the first three metrics to the final metric, it is evident that the selected measure terminates prematurely by some margin (two million iterations in case three versus 25 million iterations in the final case). This discrepancy and sensitivity are important considerations when assessing the requirements of a numerical method for the solution of an equation which exhibits slowing temporal evolutions.

Moreover, the importance of such analysis is emphasized in the cases where no exact solution is known. In the absence of an exact solution, a naive choice of convergence criteria, in spite of a highly accurate, stable numerical method, can lead to erroneous results with no problematic behaviour in solution dynamics.

## 7. Conclusions

In this work, we have provided a semi-analytical framework for computing the critical value of δ in slab geometry to arbitrary precision of the FKPDE which is a key parameter in understanding the modelling process of the equation. This framework circumvents the need to simulate the differential equation directly but does rely on the known steady-state solution. This analysis aids the subsequent numerical experimentation around the “subjectivity” of a convergence criteria.

Moreover, this work explores the sensitivity of numerical simulations of the Frank–Kamenetskii equation in all three geometries and has shown that a poor choice of termination criteria in the time-dependent simulation can lead to erroneous results which have given rise to discrepancies in the results obtained in previous studies. The numerical solutions obtained are based on a fourth-order spatial discretisation Crank–Nicolson methodology which was selected from a variety of methodologies (discussed in Section 2) due to its stability properties as well as its purported accuracy of $O(\Delta t^{2}+\Delta x^{4})$.

While this work is concerned with the dynamics of the Frank–Kamenetskii equation, the analysis is applicable across a broad range of models which employ balanced forces in the limit and sensitivity to critical parameter choices. Many problems, for which an exact steady solution is intractable, require careful consideration of whether the chosen termination criterion of the numerical method is appropriate. Such is the purpose of this work: to highlight the need for this analysis which has been overlooked and can be detrimental to the obtained numerical solution with no obvious indication of erroneous behaviour.

## Figures and Tables

**Table 1:** Results for Metric 1 (Equation (40)) with k=0.

Tolerance	$u_{0}$	$∥u^{n+1}-u^{n}∥{}_{\infty }$	Iterations	Time (*s*)	$\left|u_{0}-U\left(x_{0}\right)\right|$
1 × 10${}^{-2}$	0.0043874600317457	0.0043874600317457	1	0.016505	1.1824546923854395
1 × 10${}^{-3}$	0.7259228508482415	0.0009980375589045	306	0.778124	0.4609193015689438
1 × 10${}^{-4}$	1.04754932526698	0.0000998688171865	1309	3.431744	0.1392928271502052
1 × 10${}^{-5}$	1.1433834408127137	0.0000099999765113	4327	12.726793	0.0434587116044716
1 × 10${}^{-6}$	1.1731611425746469	0.000000999860803	13,730	41.313124	0.0136810098425384
1 × 10${}^{-7}$	1.1825216763129227	0.0000000999972707	43,319	131.283989	0.0043204761042626
1 × 10${}^{-8}$	1.1854765424428824	0.0000000099999582	136,748	421.388073	0.0013656099743029

**Table 2:** Results for Metric 2 (Equation (43)) with k=0.

Tolerance	$u_{0}$	${∥3u^{j+1}+10u^{j}-18u^{j-1}+6u^{j-2}-u^{j-3}∥}_{\infty }$	Iterations	Time (*s*)	$\left|u_{0}-U\left(x_{0}\right)\right|$
1 × 10${}^{-1}$	0.0176809250757186	0.0177620252064646	4	0.013679	1.1691612273414667
1 × 10${}^{-2}$	0.4306118298439904	0.0099551306456108	119	0.334881	0.7562303225731948
1 × 10${}^{-3}$	0.9639493976539579	0.0009994491779165	780	2.240634	0.2228927547632273
1 × 10${}^{-4}$	1.1179002770384985	0.0000999407915656	2715	7.833146	0.0689418753786868
1 × 10${}^{-5}$	1.16518464699603	0.0000099998681407	8685	25.138477	0.0216575054211552
1 × 10${}^{-6}$	1.1800085522485801	0.0000009999309731	27,424	79.867957	0.0068336001686051
1 × 10${}^{-7}$	1.1846826423441905	0.0000000999986915	86,537	253.745969	0.0021595100729948
1 × 10${}^{-8}$	1.1861595018224382	0.0000000099998776	273,340	818.690676	0.000682650594747

**Table 3:** Results for Metric 3 (Equation (44)) with k=0.

Tolerance	$u_{0}$	$∥Au^{j+1}+\delta \exp\left(u^{j+1}\right)∥{}_{\infty }$	Iterations	Time (*s*)	$\left|u_{0}-U\left(x_{0}\right)\right|$
1 × 10${}^{0}$	0	0.8784576797812904	1	0.00703	1.1824546923854395
1 × 10${}^{-1}$	0.8671871258999417	0.099945180391428	507	1.295386	0.3191571600620311
1 × 10${}^{-2}$	1.0891373563904674	0.0099978336196593	1900	4.857054	0.0976551060953823
1 × 10${}^{-3}$	1.1562735940619655	0.0009997752488236	6157	15.657636	0.0305635933242641
1 × 10${}^{-4}$	1.1772069988893805	0.0000999983696404	19,473	49.468654	0.0096346570472838
1 × 10${}^{-5}$	1.1837985277153742	0.0000099997357723	61,442	155.817235	0.0030435750581672
1 × 10${}^{-6}$	1.185880072941418	0.0000009999962707	194,022	493.842352	0.0009620745115373
1 × 10${}^{-7}$	1.1865382481444393	0.0000000999989287	613,443	1579.535638	0.0003039037766002
1 × 10${}^{-8}$	1.186746978875465	0.0000000099989235	1,949,245	5103.593224	0.0000951734923882

**Table 4:** Results for Metric 4 (Equation (45)) with k=0.

Tolerance	$u_{0}$	$∥u^{j+1}-U\left(x\right)∥{}_{\infty }$	Iterations	Time (*s*)	$\left|u_{0}-U\left(x_{0}\right)\right|$
1 × 10${}^{1}$	0.0043874600317457	1.1824546923854395	1	0.052905	1.1824546923854395
1 × 10${}^{0}$	0.1898808073704689	0.9969613450467163	43	0.28456	0.9969613450467163
1 × 10${}^{-1}$	1.086848559616201	0.0999935928009843	1854	9.863051	0.0999935928009843
1 × 10${}^{-2}$	1.176842182810891	0.0099999696062942	18,764	102.587598	0.0099999696062942
1 × 10${}^{-3}$	1.1858421532127996	0.0009999992043856	186,672	1035.636162	0.0009999992043856
1 × 10${}^{-4}$	1.1867421524649948	0.0000999999521905	1,856,056	10,066.795436	0.0000999999521905
1 × 10${}^{-5}$	1.1868321524178036	0.0000099999993817	13,781,106	73,275.25889	0.0000099999993817
1 × 10${}^{-6}$	1.1868411524178	0.0000009999993853	23,774,354	126,994.25445	0.0000009999993853
1 × 10${}^{-7}$	1.1868420524178678	0.0000000999993175	25.010,308	133,579.341297	0.0000000999993175
1 × 10${}^{-8}$	1.1868421424176347	0.0000000099995505	25,134,252	134,246.804208	0.0000000099995505

**Table 5:** Results for Metric 1 (Equation (40)) with k=1.

Tolerance	$u_{0}$	$∥u^{n+1}-u^{n}∥{}_{\infty }$	Iterations	Time (*s*)	$\left|u_{0}-U\left(x_{0}\right)\right|$
1 × 10${}^{-2}$	0.0019989994998333	0.0019989994998333	1	0.069198	1.3842953616200573
1 × 10${}^{-3}$	0.6395487028128094	0.0009979494052316	386	7.658018	0.7467456583070812
1 × 10${}^{-4}$	1.1658173814634103	0.0000999281527552	2013	38.171823	0.2204769796564803
1 × 10${}^{-5}$	1.318104044690712	0.000009997448752	6794	127.916809	0.0681903164291786
1 × 10${}^{-6}$	1.3649579014399897	0.0000009999822645	21,585	404.093311	0.0213364596799008
1 × 10${}^{-7}$	1.3798124338395485	0.0000000999971967	68,826	1284.904066	0.006481927280342
1 × 10${}^{-8}$	1.385329143692565	0.0000000099999813	264,083	4963.801774	0.0009652174273256

**Table 6:** Results for Metric 2 (Equation (43)) with k=1.

Tolerance	$u_{0}$	${∥3u^{j+1}+10u^{j}-18u^{j-1}+6u^{j-2}-u^{j-3}∥}_{\infty }$	Iterations	Time (*s*)	$\left|u_{0}-U\left(x_{0}\right)\right|$
1 × 10${}^{-2}$	0.0080230817979155	0.0080398169656547	4	0.394656	1.3782712793219751
1 × 10${}^{-3}$	1.031284350197102	0.0009989561875525	1163	31.057882	0.3550100109227885
1 × 10${}^{-4}$	1.2778192422532915	0.0000999747979129	4247	99.306974	0.1084751188665991
1 × 10${}^{-5}$	1.352413638962253	0.0000099991908382	13,651	293.392059	0.0338807221576376
1 × 10${}^{-7}$	1.3757745864245259	0.0000009999730282	43,241	905.042249	0.0105197746953647
1 × 10${}^{-7}$	1.3835081009788723	0.0000000999989336	143,953	3165.272088	0.0027862601410182

**Table 7:** Results for Metric 3 (Equation (44)) with k=1.

Tolerance	$u_{0}$	$∥Au^{j+1}+\delta \exp\left(u^{j+1}\right)∥{}_{\infty }$	Iterations	Time (*s*)	$\left|u_{0}-U\left(x_{0}\right)\right|$
1 × 10${}^{1}$	0	2	1	0.053498	1.3842953616200573
1 × 10${}^{0}$	0.6385507534075778	0.9986291284289468	386	10.218707	0.7467456583070812
1 × 10${}^{1}$	1.1660169749236722	0.0999415889807489	2016	38.285456	0.2201777206632907
1 × 10${}^{-2}$	1.3182138256098237	0.0099985391878601	6806	114.989539	0.0680705727592723
1 × 10${}^{-3}$	1.3649988210791042	0.0009999563578402	21,627	348.68668	0.0212945439392203
1 × 10${}^{-4}$	1.3798265051153304	0.0000999960410857	68,968	1094.659074	0.0064677564087783
1 × 10${}^{-5}$	1.3853407350906892	0.0000099997204215	265,246	4181.837498	0.0009536160782906

**Table 8:** Results for Metric 4 (Equation (45)) with k=1.

Tolerance	$u_{0}$	$∥u^{j+1}-U\left(x\right)∥{}_{\infty }$	Iterations	Time (*s*)	$\left|u_{0}-U\left(x_{0}\right)\right|$
1 × 10${}^{1}$	0.0019989994998333	1.3842953616200573	1	0.137639	1.3842953616200573
1 × 10${}^{0}$	0.3867396524046021	0.9995547087152885	191	8.744478	0.9995547087152885
1 × 10${}^{-1}$	1.286304734247115	0.0999909126391814	4615	185.68544	0.0999896268727756
1 × 10${}^{-2}$	1.3762969804435343	0.0099999551486123	45,436	1756.040959	0.0099973806763562
1 × 10${}^{-3}$	1.3852980074827808	0.0009999984779814	260,990	10,020.906129	0.0009963536371098
1 × 10${}^{-4}$	1.386199053950082	0.0000999964368362	364,493	13985.432266	0.0000953071698087
1 × 10${}^{-5}$	1.3862900813435808	0.000009999260675	375,948	14423.997748	0.0000042797763098

**Table 9:** Results for Metric 1 (Equation (40)) with k=2

Tolerance	$u_{0}$	$∥u^{n+1}-u^{n}∥{}_{\infty }$	Iterations	Time (*s*)	$\left|u_{0}-U\left(x_{0}\right)\right|$
1 × 10${}^{-2}$	0.0066329302180591	0.0066329302180591	1	0.023845	1.600867069781941
1 × 10${}^{-3}$	1.1878009150723405	0.0009997973665503	348	2.408809	0.4196990849276594
1 × 10${}^{-4}$	1.4800185106183377	0.0000998467765181	1261	10.935002	0.1274814893816623
1 × 10${}^{-5}$	1.5676568672606124	0.0000099977994945	4023	43.873035	0.0398431327393876
1 × 10${}^{-6}$	1.594932628198226	0.0000009998619381	12,638	149.431838	0.012567371801774
1 × 10${}^{-7}$	1.6035094558241827	0.0000000999997836	39,746	490.091531	0.0039905441758172
1 × 10${}^{-8}$	1.6062092210169532	0.0000000099998967	124,969	1568.055187	0.0012907789830467

**Table 10:** Results for Metric 2 (Equation (43)) with k=2.

Tolerance	$u_{0}$	${∥3u^{j+1}+10u^{j}-18u^{j-1}+6u^{j-2}-u^{j-3}∥}_{\infty }$	Iterations	Time (*s*)	$\left|u_{0}-U\left(x_{0}\right)\right|$
1 × 10${}^{-1}$	0.0268327806883092	0.0270192334996915	4	0.174	1.5806672193116906
1 × 10${}^{-2}$	0.9240264242568649	0.0099798818128828	181	2.386968	0.683473575743135
1 × 10${}^{-3}$	1.4039421759411046	0.0009978849204537	779	8.84406	0.2035578240588953
1 × 10${}^{-4}$	1.5443061235656819	0.0000999958083931	2546	30.141354	0.0631938764343181
1 × 10${}^{-5}$	1.5876234790181223	0.0000099999500514	8015	103.586364	0.0198765209818776
1 × 10${}^{-6}$	1.6012072302501235	0.000000999980045	25,186	337.728205	0.0062927697498765
1 × 10${}^{-7}$	1.6054862757331536	0.0000000999986498	79,267	1083.028543	0.0020137242668463
1 × 10${}^{-8}$	1.6068224310925365	0.0000000099999726	247,196	3402.854252	0.0006775689074634

**Table 11:** Results for Metric 3 (Equation (44)) with k=2.

Tolerance	$u_{0}$	$∥Au^{j+1}+\delta \exp\left(u^{j+1}\right)∥{}_{\infty }$	Iterations	Time (*s*)	$\left|u_{0}-U\left(x_{0}\right)\right|$
1 × 10${}^{1}$	0	3.3219921	1	0.024878	1.600867069781941
1 × 10${}^{0}$	1.001655478031055	0.9947901931747936	217	2.023518	0.6038668179551216
1 × 10${}^{-1}$	1.4267779133580645	0.0998792820920134	883	7.71426	0.1805248451118036
1 × 10${}^{-2}$	1.5514480665764723	0.0099936577707123	2869	24.823671	0.0560322477020716
1 × 10${}^{-3}$	1.5898637256230181	0.0009999961587575	9026	101.042866	0.0176343062204019
1 × 10${}^{-4}$	1.6019140522787323	0.0000999974878617	28,374	358.878846	0.0055857509606889
1 × 10${}^{-5}$	1.60570874136426	0.0000099997830603	89,299	1168.869368	0.0017912389619574
1 × 10${}^{-6}$	1.6068904477799801	0.0000009999726736	277,889	3676.908969	0.0006095502564198
1 × 10${}^{-7}$	1.607222162512687	0.0000000999824756	785,881	10,427.140501	0.0002778372938181
1 × 10${}^{-8}$	1.6072854711405922	0.0000000099049089	1,742,919	23,081.90619	0.0002145288442714
